# Transcriptome analysis reveals the prognostic and immune infiltration characteristics of glycolysis and hypoxia in head and neck squamous cell carcinoma

**DOI:** 10.1186/s12885-022-09449-9

**Published:** 2022-03-31

**Authors:** Jun Liu, Jianjun Lu, Wenli Li

**Affiliations:** 1 Reproductive Medicine Center, Yue Bei People’s Hospital, Shantou University Medical College, 133 Huimin South Road, Shaoguan, 512025 China; 2 Medical Research Center, Yue Bei People’s Hospital, Shantou University Medical College, Shaoguan, 512025 China; 3 Department of Medical Affairs, First Affiliated Hospital of Sun Yat-Sen University, Guangzhou, 510080 China; 4 The Second School of Clinical Medicine, Southern Medical University, Guangzhou, 510080 China

**Keywords:** Head and neck squamous cell carcinoma, Overall survival, Glycolysis, Hypoxia, Gene signature

## Abstract

**Background:**

This study aims to construct a new prognostic gene signature in survival prediction and risk stratification for patients with Head and neck squamous cell carcinoma (HNSCC).

**Method:**

The transcriptome profiling data and hallmark gene sets in the Molecular Signatures Database was used to explore the cancer hallmarks most relevant to the prognosis of HNSCC patients. Differential gene expression analysis, weighted gene co-expression network analysis, univariate COX regression analysis, random forest algorithm and multiple combinatorial screening were used to construct the prognostic gene signature. The predictive ability of gene signature was verified in the TCGA HNSCC cohort as the training set and the GEO HNSCC cohorts (GSE41613 and GSE42743) as the validation sets, respectively. Moreover, the correlations between risk scores and immune infiltration patterns, as well as risk scores and genomic changes were explored.

**Results:**

A total of 3391 differentially expressed genes in HNSCC were screened. Glycolysis and hypoxia were screened as the main risk factors for OS in HNSCC. Using univariate Cox analysis, 97 prognostic candidates were identified (*P* < 0.05). Top 10 important genes were then screened out by random forest. Using multiple combinatorial screening, a combination with less genes and more significant P value was used to construct the prognostic gene signature (RNF144A, STC1, P4HA1, FMNL3, ANO1, BASP1, MME, PLEKHG2 and DKK1). Kaplan–Meier analysis showed that patients with higher risk scores had worse overall survival (*p* < 0.001). The ROC curve showed that the risk score had a good predictive efficiency (AUC > 0.66). Subsequently, the predictive ability of the risk score was verified in the validation sets. Moreover, the two-factor survival analysis combining the cancer hallmarks and risk scores suggested that HNSCC patients with the high hypoxia or glycolysis & high risk-score showed the worst prognosis. Besides, a nomogram based on the nine-gene signature was established for clinical practice. Furthermore, the risk score was significantly related to tumor immune infiltration profiles and genome changes.

**Conclusion:**

This nine-gene signature associated with glycolysis and hypoxia can not only be used for prognosis prediction and risk stratification, but also may be a potential therapeutic target for patients with HNSCC.

**Supplementary Information:**

The online version contains supplementary material available at 10.1186/s12885-022-09449-9.

## Introduction

Head and neck squamous cell carcinoma (HNSCC), which includes a group of heterogeneous tumors from the squamous epithelium of the oral cavity, oropharynx, larynx and hypopharynx, is the seventh most common cancer in the world [1]. There are approximately 645,000 new cases of HNSCC each year worldwide. After receiving aggressive therapy, the 5-year survival rate of patients with HNSCC is still less than 50%. Patients with recurrent or metastatic HNSCC usually have a poor prognosis, with a median overall survival (OS) of only about 6 months. As far as we know, smoking, drinking, and human papilloma virus infection are risk factors for HNSCC. Moreover, the site of HPV infection is related to OS of HNSCC patients. For example, HPV positivity in the oropharynx, hypopharynx, oral cavity, and larynx is associated with improved OS, while HPV positivity in the nasopharynx and sinus passages is not associated with OS [2]. In the treatment of HNSCC, TNM staging is routinely used as the basis for selecting an appropriate treatment plan including surgery, radiotherapy and/or chemotherapy. Surgery is used for HNSCC patients whose tumors have not spread. Radiation therapy can be used for HNSCC patients with advanced pathological stage. Recent studies have shown that simultaneous radiotherapy and chemotherapy can improve patient survival, but it is not tolerated by many patients. Thus, although there have been some improvements in the treatment of HNSCC in the past few decades, the OS rate has not been improved significantly, which is mainly related to advanced stage at the time of diagnosis and the high treatment failure rate of advanced stage. Therefore, screening new prognostic biomarkers is essential to improve the clinical efficacy and OS of patients with HNSCC [3].

The hallmarks of cancer can show the basic characteristics of tumor cells. Recent studies suggested that some cancer hallmarks such as glycolysis and hypoxia were significantly related to the prognosis of patients with HNSCC. Glycolysis as the preferred pathway of energy metabolism is a characteristic of cancer cells. Therefore, cancer cells often exhibit increased glycolysis [4]. Previous studies have suggested that in HPV-negative HNSCC, the expression of genes related to glycolysis increased [5]. And glycolysis can be used as a biomarker to predict the prognosis of HNSCC patients [6]. In addition, pyruvate metabolism, a major glycolytic metabolite, was considered a potential anti-cancer target [7]. In terms of intratumoral hypoxia, tumor hypoxia is related to chemotherapy resistance and radiotherapy response in HNSCC [8, 9]. Moreover, hypoxia-inducible factor-1α was involved in intratumoral hypoxia-mediated tumor metastasis in HNSCC [10]. As hypoxic cells were more resistant to treatment, intratumoral hypoxia was an indicator of poor prognosis in HNSCC [11, 12]. Therefore, certain genes related to cancer hallmarks such as glycolysis and hypoxia are expected to serve as prognostic biomarkers for HNSCC.

Currently, treatment decisions for individual HNSCC patients are mainly based on the patient’s specific condition. However, the predictive ability and accuracy of traditional pathological staging have shown some deficiencies. Therefore, there is an urgent need to find new accurate and reliable predictors for HNSCC to guide risk stratification management and develop personalized treatment plans. In this study, we extracted an HNSCC cohort from the Cancer Genome Atlas (TCGA). Then, we explored the correlations between the cancer hallmarks and HNSCC. Next, we screened important prognostic genes related to glycolysis and hypoxia. A gene signature for survival prediction was then established. Subsequently, the prognostic value of the risk score based on gene signature was verified in the training set from TCGA and the validation set from the Gene Expression Omnibus (GEO). In addition, the correlations between risk scores and immune cell infiltration patterns, immune-related molecules and genomic changes were explored, respectively. Time-dependent receiver operating characteristic (t-ROC) curve was used to verify the prediction accuracy of the survival model. Taken together, this study comprehensively analyzed the prognostic value of a new gene signature related to cancer hallmarks including glycolysis and hypoxia in HNSCC. This gene signature can be used not only as a prognostic biomarker, but also as a potential therapeutic target for HNSCC.

## Material and methods

### Data set preparation and data processing

The training dataset and validation dataset for constructing prognostic gene signature were download from the TCGA and GEO databases, respectively. The training dataset with HNSCC-mRNA expression profile and clinical information obtained from the TCGA database (http://cancergenome.nih.gov/) included 546 HNSCC patients. The validation data sets (GSE41613 and GSE42743) with HNSCC-mRNA expression profile and clinical information downloaded from the GEO database (http://www.ncbi.nlm.nih.gov/geo/) included 97 and 74 HNSCC patients, respectively. The above two databases are publicly available. Therefore, this study did not require the approval of the local ethics committee.

In order to make the gene expression profiles of different platforms comparable, we downloaded the data in FPKM format from the TCGA database and converted it into TPM format. Figure S1 showed the sample-normalized boxplots of two HNSCC cohorts from the GEO database. Subsequently, we performed a log2 normalization conversion on the above data. Meanwhile, using the limma package in R, the chip data downloaded from GEO was normalized [13]. Subsequently, 498 HNSCC samples with follow-up information and overall survival greater than zero from the TCGA RNAseq data were screened. The gene expression profile was obtained by removing the genes with zero expression levels in 50% of the samples. Meanwhile, the expression profile of the immune-related gene set was extracted. On the other hand, we performed the background correction on the gene chip data from GEO database. Then, 97 and 74 HNSCC samples with follow-up information and overall survival greater than zero from GSE41613 and GSE42743 were screened, respectively. Next, using the R package GEOquery, the chip probes were mapped to GeneSymbol. Finally, we get the gene expression profile by removing the probes mapped to multiple genes and taking the median of the multiple probes mapped to a single gene [13].

### Selection of candidate genes and establishment of a gene signature

Based on the transcriptome profiling data and hallmark gene sets from the Molecular Signatures Database (MSigDB), the single-sample gene set enrichment analysis (ssGSEA) algorithm (R package “gsva”) was used to quantify the performance of cancer hallmarks in the training set [14, 15]. The R package “survival” was used to perform univariate Cox proportional hazards regression analysis (Cox-PH) to assess the significance of various cancer hallmarks in HNSCC. The R package “wgcna” (weighted gene co-expression network analysis) was used to construct a scale-free co-expression network. Subsequently, transcriptome profiling data and ssGSEA scores were used to screen the gene modules most related to glycolysis and hypoxia [16]. Gene significance (GS) is used to quantify the correlation between individual genes and ssGSEA scores of glycolysis and hypoxia. Module member represented the correlations between module characteristic genes and gene expression profiles. Using the p-value threshold of GS < 0.0001 and the p-value of univariate Cox regression < 0.01, 97 prognostic genes most related to glycolysis and hypoxia were identified [17]. Next, random forest was used to rank the importance of genes, and the top ten important genes were selected. The gene signature with the smaller number of genes and the more significant P value was selected from multiple combinations of ten genes and used to construct a survival model.

### Survival analysis based on the risk score

The Z-score method was used to standardize the ssGSEA scores and risk scores [18]. The Kaplan–Meier method was used to perform survival analysis. The Cox proportional hazards regression model was used to evaluate the importance of each parameter to OS. Taking the median of risk scores as the cut-off value, HNSCC patients were divided into high- and low-risk groups, and the prognosis of the two groups were compared in the training set and validation set. The ROC curve was used to evaluate the prediction accuracy of the risk score. The t-ROC was used to evaluate the predictive ability (R package “survival-ROC”) [19]. Furthermore, a two-factor survival analysis combining risk score and cancer hallmarks including glycolysis and hypoxia was conducted to evaluate the impact of risk score, glycolysis and hypoxia on the prognosis of patients with HNSCC.

### Comparing the survival prediction ability among various gene signatures in HNSCC

Previous studies had constructed some gene signatures that could be used to predict the survival of HNSCC patients. For example, an eight-gene signature constructed by Baoling Liu et al. and a ten-gene signature constructed by Zhaoyi Lu et al. showed good predictive ability in HNSCC [20, 21]. Therefore, in order to further evaluate the prognostic value of the genetic features constructed in this study, we compared the predictive power of the three gene signatures in HNSCC.

### Drug susceptibility analysis of potential inhibitors of nine hub genes

To explore potential inhibitors available for nine hub genes, we performed drug sensitivity analysis. Drug sensitivity data for gene inhibitors were downloaded from the CellMiner™ database (version: 2020.3, database: 2.4.2, https://discover.nci.nih.gov/cellminer/home.do) [22]. The R packages “impute”, “limma”, “ggplot2” and “ggpubr” were used for data processing and visualization.

### Establishment and evaluation of nomogram for survival prediction in HNSCC patients

Nomogram is an effective method for predicting the prognosis of cancer patients by simplifying complex statistical prediction models into contour maps that assess the probability of individual patients’ OS [23]. In this study, we constructed a nomogram based on the nine-gene signature to assess the probability of OS for HNSCC patients at 1-, 3-, and 5-year. Meanwhile, the predicted probability of the nomogram was compared with the observed actual probability through the calibration curve to verify the accuracy of the nomogram. Overlap with the reference line indicates that the model is accurate. In addition, t-ROC analysis was used to evaluate the survival prediction ability of this nomogram.

### Correlation analysis between risk score and tumor immune microenvironment (TIME)

TIME is the immune-related complex environment in which tumor cells grow. Therefore, we conducted a correlation analysis between the risk score and TIME. Firstly, multiple analysis methods including TIMER, CIBERSORT, CIBERSORT-ABS, QUANTISEQ, MCPCOUNTER, XCELL and EPIC were used to analyze the correlations between risk scores and immune cell infiltration status [24]. The immune cells involved in the analysis included CD4 T cells, CD8 T cells, B cells, neutrophils, and so on. Subsequently, the correlations between the risk score and immune cells, and the correlations between the risk score and the expression levels of immune-related molecules were explored respectively. Furthermore, the correlations between the risk score and genomic changes including gene mutation rate, gene mutation load, gene microsatellite instability and TP53 mutation were analyzed respectively.

### Bioinformatics and statistical analysis

The hallmark gene sets of glycolysis and hypoxia from the MSigDB were used for GSEA analysis to verify the glycolysis and hypoxia status in the high-risk score group [25]. IBM SPSS Statistics 20 (IBM Corp., Armonk, NY, USA) and R software (version 3.5.2, http://www.r-project.org) were used to analyze data and draw figures. Z-scores were used to standardize ssGSEA scores and risk scores. The Kaplan–Meier method was used to draw the survival curve, and the log-rank test was used to assess the difference. The “wilcox.test” function was used to compare the risk scores between groups. The Cox proportional hazards regression model was used to evaluate the importance of each parameter to OS.

## Results

### Schematic diagram of research design

**Fig. 1:**
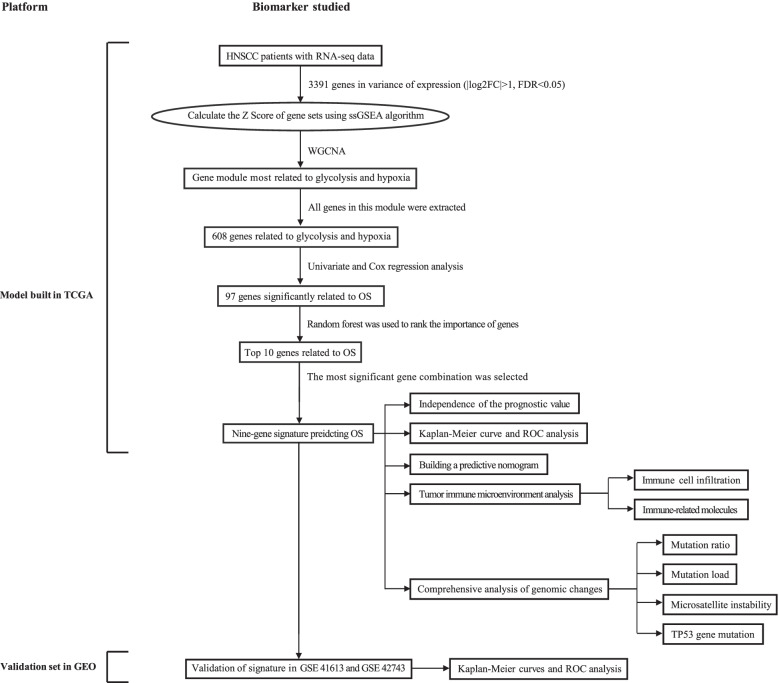
Overall flowchart of this study. LASSO, least absolute shrinkage and selection operator; HNSCC, head and neck squamous cell carcinoma; ssGSEA, single-sample gene set enrichment analysis; ROC, receiver operating characteristic; WGCNA, weighted gene co-expression network analysis

Figure 1 is a flowchart of the entire work of this research. The detailed process of constructing the OS prediction model for HNSCC patients is as follows. Firstly, glycolysis and hypoxia were identified as the main risk factors for the OS of patients with HNSCC among virous cancer hallmarks. Then, ssGSEA algorithm, WGCNA and univariate Cox regression analysis were used to screen promising candidates. Next, random forest algorithm and multiple combined screening methods were used to establish prognostic gene signature related to glycolysis and hypoxia. Finally, the prognostic value of the risk score based on the gene signature was evaluated in the training set and two independent validation sets (GSEGSE41613 and GSE42743). The patients’ information in the TCGA and GEO cohorts is shown in Table 1.

**Table 1:** Clinical information of patients with HNSCC included in this study

Characteristic		TCGA(*n* = 498)	GSE41613(*n* = 97)	GSE42743(*n* = 74)
Status	Survival	281(56.4%)	46(47.5%)	51(68.9%)
	Dead	217(43.5%)	51(52.5%)	23(31.1%)
Age	< 60	220(44.2%)	50(51.6%)	37(50%)
	> = 60	278(55.8%)	47(48.4%)	37(50%)
Gender	Female	133(26.7%)	31(31.9%)	16(21.6%)
	Male	365(73.3%)	66(68.1%)	58(78.4%)
T	T1	33(6.6%)		
	T2	141(28.3%)		
	T3	130(26.1%)		
	T4	179(36%)		
	TX	11(2.2%)		
	NA	4(0.8%)		
M	M0	468(94%)		
	M1	5(1%)		
	MX	20(4%)		
	NA	5(1%)		
N	N0	237(47.5%)		
	N1	80(16%)		
	N2	152(30.5%)		
	N3	7(1.4%)		
	NX	18(3.6%)		
	NA	4(1%)		
Stage	Stage I	19(3.8%)	41(42.3%)	3(4.1%)
	Stage II	93(18.6%)		27(36.5%)
	Stage III	102(20.4%)	56(57.7%)	28(37.8%)
	Stage IV	270(57.2%)		16(21.6%)
	NA	14(2.8%)		
Grade	G1	61(12.2%)		
	G2	298(59.9%)		
	G3	119(23.9%)		
	G4	2(0.4%)		
	GX	15(3%)		
	NA	3(0.6%)		

### Glycolysis and hypoxia were identified as the main risk factors for the OS of patients with HNSCC

**Fig. 2:**
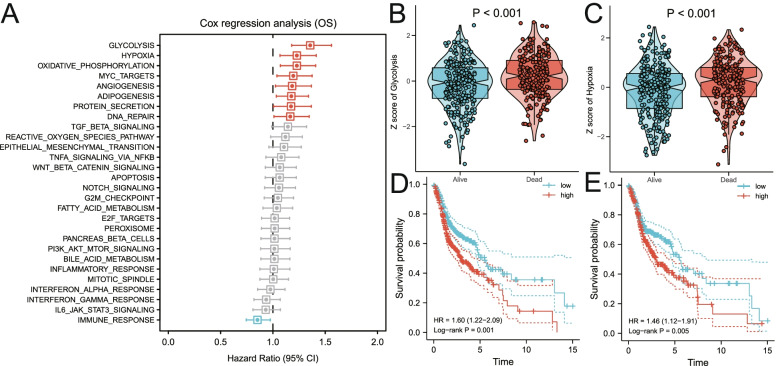
Glycolysis and hypoxia are the main risk factors for the overall survival of patients with HNSCC. **A** Univariate Cox regression analysis showed that glycolysis and hypoxia were the main risk factors in various cancer hallmarks. **B-C** The Z Score values of patients who died during the follow-up period were significantly higher than those of alive patients during the follow-up period in glycolysis and hypoxia. **D-E** Kaplan–Meier analysis suggested that patients with higher Z scores exhibited poorer OS in glycolysis and hypoxia. OS, overall survival; Pl3-Akt-mTOR, phosphatidylinositol-3-kinase-Akt-mammalian target of rapamycin; TGF, transforming growth factor

According to the ssGSEA scores and OS information of cancer hallmarks in the training set, the Cox coefficient of each marker was calculated and sorted. The results of univariate analysis suggested that glycolysis and hypoxia have the greatest impact on the survival of patients with HNSCC compared with other cancer hallmarks including oxidative phosphorylation, MYC_targets, angiogenesis, adipogenesis, protein secretion and DNA repair (Fig. 2A). As shown in Fig. 2B-C, the ssGSEA Z-scores of glycolysis and hypoxic were higher in the patients who died during the follow-up period compared with that of alive patients. Subsequently, using the median of Z-scores as the cut-off value, 498 HNSCC patients in the training set were divided into high- and low-risk groups. The OS rate of the high glycolytic Z-scores group was lower than that of the low glycolytic Z-scores group (HR = 1.60, *P* = 0.001; Fig. 2D). Meanwhile, the OS rate of the high-hypoxic Z-score group was lower than that of the low-hypoxic Z-score group (HR = 1.46, *P* = 0.005; Fig. 2E).

### Establishment of prognostic gene signature related to glycolysis and hypoxia

**Fig. 3:**
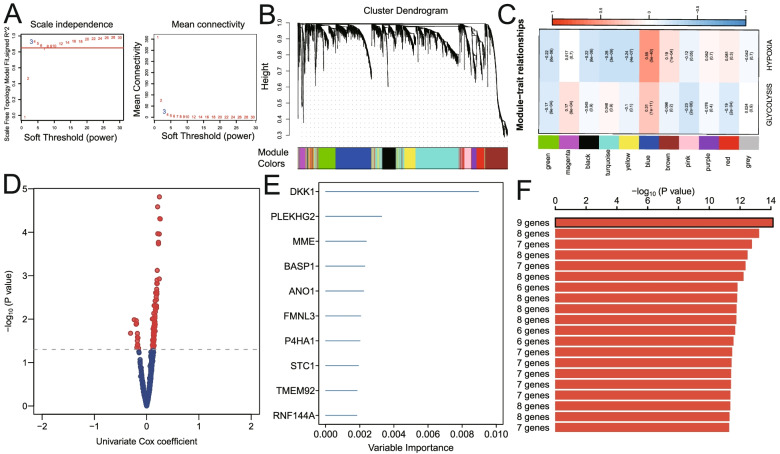
Establishment of a gene signature related to glycolysis and hypoxia. **A** Identification of DEGs in HNSCC. Using |log2FC|> 1 and FDR < 0.05, a total of 3391 DEGs in HNSCC were identified. **B** 3391 DEGs were used to construct the WGCNA network to identify non-grey modules. **C** Construction of co-expression modules related to key cancer hallmarks. The blue module was identified as the module with higher correlation with glycolysis and hypoxia (*r* > 0.3, *P* < 0.0001). **D** Using univariate Cox analysis, 97 candidates related to the prognosis of patients with HNSCC were identified from the genes of the blue module (*P* < 0.05). **E** Using random forest, the top 10 genes with the highest gene importance were screened out. **F** The gene combination with a relatively small number of genes and a relatively significant P value was selected from the multiple combinations of 10 genes to construct a survival prediction model. DEGs, differentially expressed genes. FDR, false discovery rate. WGCNA, weighted gene co-expression network analysis

Using |log2FC|> 1 and FDR < 0.05, a total of 3391 DEGs in HNSCC were identified (Fig. 3A). WGCNA was conducted using transcriptome profiling data of 3391 genes and ssGSEAZ scores of the two cancer hallmarks (including glycolysis and hypoxia) in the training set. Taking β = 3 as the optimal soft threshold to ensure the scale-free co-expression of the network, the non-gray modules were generated (Fig. 3B). Then co-expression modules related to key cancer hallmarks were constructed. And the blue module was identified as the module with higher correlation with glycolysis and hypoxia (*r* > 0.3, *P* < 0.0001; Fig. 3C). Using < 0.01 as the threshold of the P value for univariate Cox regression, 97 genes from the blue module were identified as promising candidates related to the prognosis of patients with HNSCC (Fig. 3D). Next, random forest was used to rank the importance of genes, and the top 10 relatively important genes were screened out (Fig. 3E). Subsequently, the gene combination with a smaller number of genes and a more significant P value was selected from multiple combinations of 10 genes to construct a survival risk model (Fig. 3F). Finally, nine hub genes were used to construct a prognostic model for patients with HNSCC: risk score = (-0.055) * RNF144A + 0.011 * STC1 + 0.016 * P4HA1 + (-0.042) * FMNL3 + 0.002 * ANO1 + 0.002 * BASP1 + 0.004MME + (-0.106) * PLEKHG2 + 0.003 * DKK1.

### Risk score was an independent risk factor for OS in the training set

**Fig. 4:**
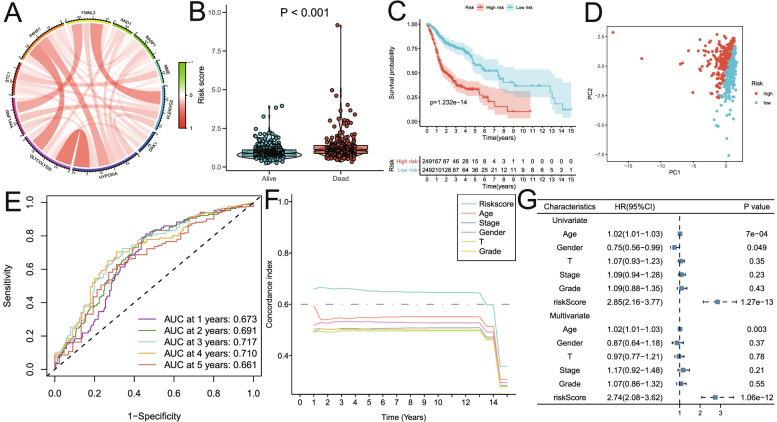
The risk score predicts poor survival in the training set. **A** Correlation analysis between expression levels of nine hub genes and Z-scores of key cancer hallmarks (including glycolysis and hypoxia) in the training set. **B** Patients who died during follow-up had a higher risk score than those who were alive. **C** Kaplan–Meier analysis showed that the high-risk score group exhibited worse overall survival. **D** Principal component analysis suggested that risk score could be used as a new dimension to evaluate the prognosis of HNSCC patients. **E** The ROC curve showed the prediction efficiency of the risk score for the survival rate in the training set (AUC > 0.66). **F** The tROC analysis showed that the predictive power of risk score was significantly higher than that of other clinical characteristics. **G** Univariate and multivariate Cox regression analysis showed that risk score was an independent risk factor for OS in patients with HNSCC. HR, hazard ratio; OS, overall survival; tROC, time-dependent receiver operating characteristics

Correlation analysis suggested the expression levels of nine hub genes were related with the Z-scores of key cancer hallmarks (including glycolysis and hypoxia) in the training set (Fig. 4A). The risk scores of the patients who died during the follow-up period were significantly higher than that of alive patients (Fig. 4B). Kaplan–Meier analysis showed that the high-risk score group exhibited worse overall survival (*P* < 0.001, Fig. 4C). Principal component analysis suggested that risk score could be used as a new dimension to assess the prognosis of HNSCC patients (Fig. 4D). The ROC curve showed that the AUCs of risk scores for predicting 1-, 2-, 3-, 4-, and 5-year survival rates were 0.673, 0.691, 0.717, 0.710, and 0.661, respectively, suggesting that risk score was a good model for predicting the survival of HNSCC patients (Fig. 4E). Subsequent tROC analysis showed that the survival predictive power of the risk score was better than that of other clinicopathological characters (Fig. 4F). Furthermore, univariate and multivariate Cox regression analysis showed that the risk score (HR = 1.33, *p* < 0.001), N stage (HR = 1.24, *p* < 0.01), M stage (HR = 4.25, *p* < 0.01) and age (HR = 1.02, *p* < 0.001) were independent risk factors affecting OS of HNSCC patients (Fig. 4G).

### Validating the prognostic values of the nine-gene signature in independent HNSCC datasets

**Fig. 5:**
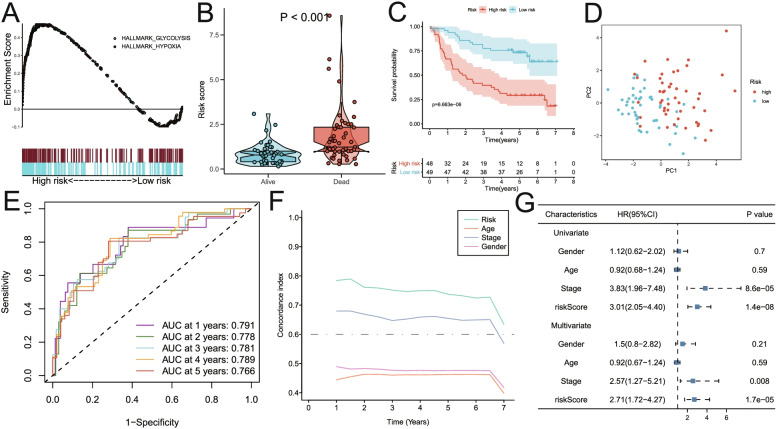
Validation of the risk model in the GSE41613 dataset. **A** GSEA analysis showed that glycolysis and hypoxia pathways were significantly enriched in the high-risk group. **B** The risk score of patients who died during follow-up was higher than that of alive patients. **C** Kaplan–Meier analysis showed that patients with higher risk scores had worse overall survival. **D** Principal component analysis suggested that risk score could be used as a new dimension to evaluate the prognosis of HNSCC patients. **E** The ROC curve showed the prediction efficiency of the risk score in the validation dataset for the survival rate of patients with HNSCC (AUC > 0.76). **F** The tROC analysis showed that the survival predictive ability of risk score was significantly higher than that of other clinical features. **G** Univariate and multivariate Cox regression analysis showed that risk score was an independent risk factor for PFS in patients with HNSCC. HR, hazard ratio; PFS, progression-free survival; tROC, time-dependent receiver operating characteristics

In order to confirm the robustness of the prognostic nine-gene signature related to glycolysis and hypoxia, two independent external cohorts (GSE41613 and GSE42743) from the GEO database were used as validation sets. The analysis results in the GSE41613 HNSCC cohort were shown in Fig. 5. Glycolysis and hypoxia pathways were significantly enriched in the high-risk group (Fig. 5A). Meanwhile, the risk score of patients who died during the follow-up period was significantly higher than that of alive patients (*P* < 0.0001; Fig. 5B). Kaplan–Meier analysis showed that patients with high-risk scores had worse OS than those with low-risk scores (*p* < 0.001; Fig. 5C). Next, the principal component analysis suggested that risk score could be used as a new dimension to assess the prognosis of HNSCC patients (Fig. 5D). The ROC curve showed that AUCs of risk scores for predicting 1-, 2-, 3-, 4-, and 5-year survival rates were 0.791, 0.778, 0.781, 0.789, and 0.766, respectively, indicating that the risk score was a good predictive model for HNSCC patients (Fig. 5E). The tROC analysis showed that the survival predictive ability of the risk score was significantly higher than other clinicopathological characteristics (Fig. 5F). In addition, multivariate Cox regression analysis suggested that risk score was an independent risk factor for PFS in HNSCC patients (HR = 2.71, *p* < 0.001; Fig. 5G).

**Fig. 6:**
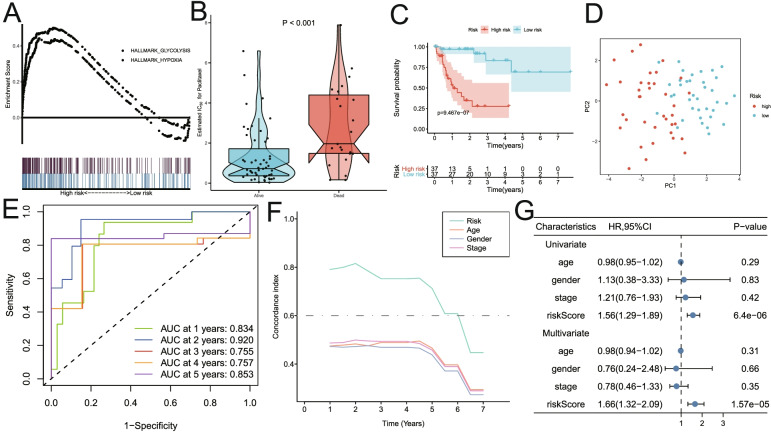
Validation of the risk model in the GSE42743 dataset. **A** GSEA analysis showed that glycolysis and hypoxia pathways were significantly enriched in the high-risk group. **B** The risk score of patients who died during follow-up was higher than that of alive patients. **C** Kaplan–Meier analysis showed that patients with higher risk scores had worse overall survival. **D** Principal component analysis suggested that risk score could be used as a new dimension to evaluate the prognosis of HNSCC patients. **E** The ROC curve showed the prediction efficiency of the risk score in the validation dataset for the survival rate of patients with HNSCC (AUC > 0.75). **F** The tROC analysis showed that the survival predictive ability of risk score was significantly higher than that of other clinical features. **G** Univariate and multivariate Cox regression analysis showed that risk score was an independent risk factor for PFS in patients with HNSCC. HR, hazard ratio; PFS, progression-free survival; tROC, time-dependent receiver operating characteristics

Similarly, the analysis results in the GSE42743 HNSCC cohort were shown in Fig. 6. Glycolysis and hypoxia pathways were significantly enriched in the high-risk group (Fig. 6A). The risk score of patients who died during the follow-up period was significantly higher than that of alive patients (*P* < 0.0001; Fig. 6B). Kaplan–Meier analysis showed that patients with high-risk scores had worse OS than those with low-risk scores (*p* < 0.001; Fig. 6C). Principal component analysis suggested that risk score could be used as a new dimension to assess the prognosis of HNSCC patients (Fig. 6D). The ROC curve showed that AUCs of risk scores for predicting 1-, 2-, 3-, 4-, and 5-year survival rates were 0.834, 0.920, 0.755, 0.757, and 0.853, respectively, indicating that the risk score was a good predictive model for HNSCC patients (Fig. 6E). The tROC analysis showed that the survival predictive ability of risk score was better than other clinicopathological characteristics (Fig. 6F). Moreover, multivariate Cox regression analysis showed that risk score was an independent risk factor for PFS in HNSCC patients (HR = 1.66, *p* < 0.001; Fig. 6G).

### Correlation between risk score and cancer hallmarks and corresponding two-factor survival analysis

**Fig. 7:**
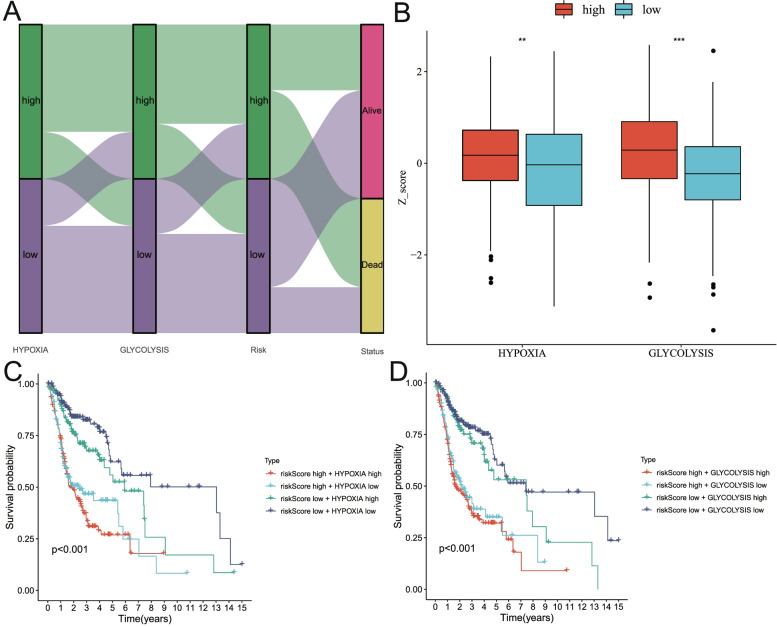
Two-factor survival analysis combining cancer hallmarks and risk scores. **A** Correlation analysis of cancer hallmarks (including hypoxia and glycolysis), risk scores and survival status of patients with HNSCC. **B** The Z-Scores of high-risk patients were significantly higher than those of low-risk patients both in hypoxia and glycolysis. **C** A two-factor survival analysis combining hypoxia and risk score suggested that high-hypoxia & high-risk score predicted a worse prognosis. **D** A two-factor survival analysis combining glycolysis and risk scores suggested that high-glycolysis & high-risk scores predicted a worse prognosis

As shown in Fig. 7A, the proportion of patients with high hypoxia, high glycolysis and high risk scores among patients who died during follow-up was higher, while the proportion of patients with low hypoxia, low glycolysis, and low risk scores among alive patients was higher. As shown in Fig. 7B, the Z-socres of hypoxia and glycolysis were both higher in patients with high-risk groups. Subsequently, a two-factor survival analysis combining risk score and cancer hallmarks suggested that HNSCC patients with low risk score & low glycolysis or hypoxia Z-socres showed the best OS, while HNSCC patients with high risk score & high glycolysis or hypoxia Z-socres showed the worst prognosis (Fig. 7C-D).

### The risk score was an indicator of poor prognosis in various subgroup cohorts

**Fig. 8:**
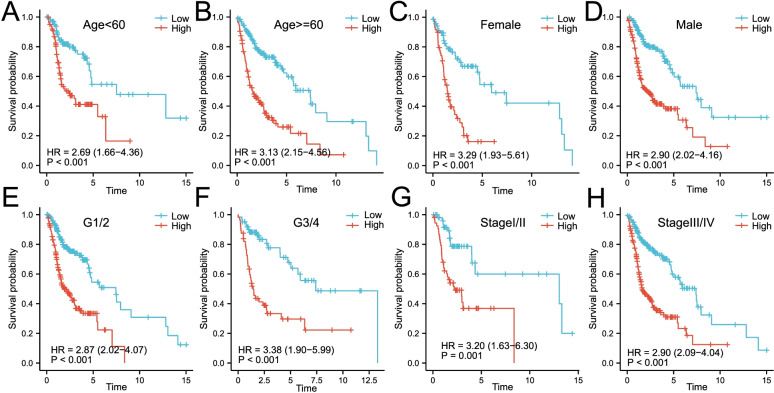
The risk score based on the nine-gene signature is a valuable marker for poor prognosis in various subgroups divided by clinicopathological characteristics. **A-H** The nine-gene signature could distinguish high-risk patients in a variety of subgroups divided by clinicopathological characteristics including age, gender, grades, and pathological stages. HR, risk ratio

As shown in Fig. 8A-H, the risk score based on the nine-gene signature could distinguish high-risk patients with poor prognosis in various subgroups divided by clinicopathological characteristics including age, gender, grades and pathological stages (*p* < 0.001). Meanwhile, we divided HNSCC patients into four subgroups based on TP53 and TTN mutational status, and then analyzed the effect of risk score on the survival of HNSCC patients within each subgroup. As shown in Figure S2, the risk score based on the nine-gene signature could distinguish patients with poor prognosis in four subgroups divided by TP53 and TTN gene mutations (*p* < 0.001).

### This nine-gene signature was a good model for predicting the survival of HNSCC patients

**Fig. 9:**
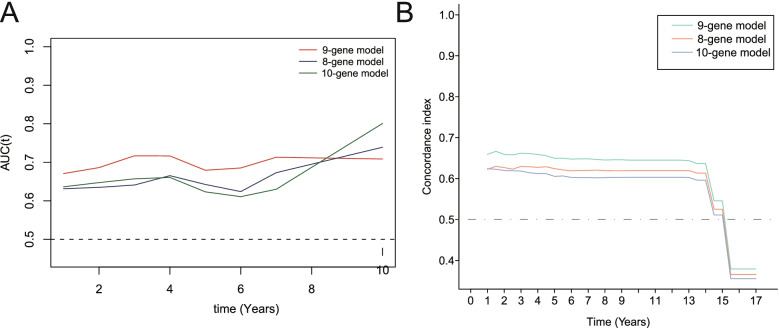
Comparison of survival prediction ability among various prognostic gene signatures in HNSCC. The tROC and C-index analyses were used to compare the predictive power of various gene signatures. Three gene signatures included were defined as follows: the nine-gene signature were constructed in our study; the eight-gene signature (CBX3, GNA12, P4HA1, PLAU, PPL, RAB25, EPHX3, and HLF) was constructed by Baoling Liu et al.; the ten-gene signature (IL2RA, CCL5, SLC2A6, PTX3, PDGFA, INHBA, HS3ST1, TGFBI, GAS7, and RAl14) was constructed by Zhaoyi Lu et al. **A** The tROC analysis showed that the predictive ability of the nine-gene signature was better than that of the other two gene signatures within eight years. **B** C-index analysis indicated that the prediction results of the nine-gene signature were basically consistent with the survival results actually observed in HNSCC

Comparing the predictive power of the nine-gene signature built in this work with that of gene signatures built by other previous studies could help to further evaluate the prognostic value of the nine-gene signature. Therefore, we included two previously established gene signatures into the analysis. Included three gene signatures were as follows: the nine-gene signature was built in this work; the eight-gene signature (CBX3, GNA12, P4HA1, PLAU, PPL, RAB25, EPHX3, and HLF) was built by Baoling Liu et al.; the ten-gene signature (IL2RA, CCL5, SLC2A6, PTX3, PDGFA, INHBA, HS3ST1, TGFB1, GAS7, and RA114) was built by Zhaoyi Lu et al. As shown in Fig. 9, the predictive ability of the nine-gene signature was better than that of the other two gene signatures within eight years. Moreover, the predicted results of the nine-gene signature were basically consistent with the survival results actually observed. These results indicated that the nine-gene signature was a good prognostic model for HNSCC patients.

### Drug susceptibility analysis of related inhibitors of nine hub genes

To explore potential inhibitors of nine hub genes, we performed a drug sensitivity analysis using the CellMiner™ database. The results showed that RNF144A expression was positively correlated with the drug sensitivity of Vemurafenib, Dabrafenib, Encorafenib and ABT-199; BASP1 expression was negatively correlated with drug sensitivity of Tamoxifen, Vemurafenib, Nilotinib and Selumetinib; BASP1 expression was positively correlated with drug sensitivity of Dasatinib. STC1 expression was positively correlated with the drug sensitivity of Olaparib, Simvastatin and Rapamycin; PLEKHG2 was negatively correlated with drug sensitivity of Palbociclib, P4HA1 was negatively correlated with drug sensitivity of 6-THIOGUANINE. DKK1 was negatively correlated with drug sensitivity of Sunitinib (Figure S3) 。

### Building a nomogram to predict OS in HNSCC patients

**Fig. 10:**
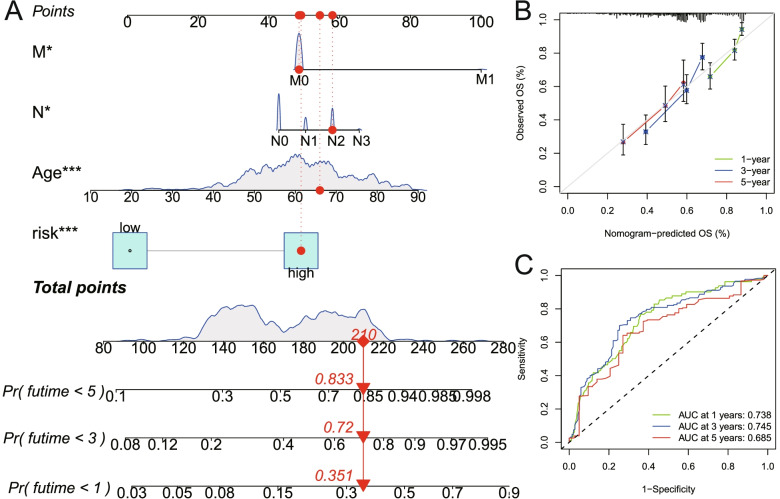
Building a nomogram based on the nine-gene signature for HNSCC patients. **A** A nomogram combining the nine-gene signature, M stage, N stage and age was constructed. **B** The calibration chart showed that the predicted 1- 3-, and 5-year survival probabilities were basically consistent with actual observations. **C** The tROC analysis indicated that this comprehensive nomogram had good survival prediction ability

Combining the nine-gene signature, patient age and pathological stage, a comprehensive nomogram that can be used in clinical practice was constructed (Fig. 10A). Subsequently, the predictive power of this nomogram was verified by the calibration curve (Fig. 10B). Moreover, the tROC curve confirmed the good survival prediction ability of this nomogram (AUC > 0.68, Fig. 10C).

### Correlation analysis between risk score and tumor immune infiltration

**Fig. 11:**
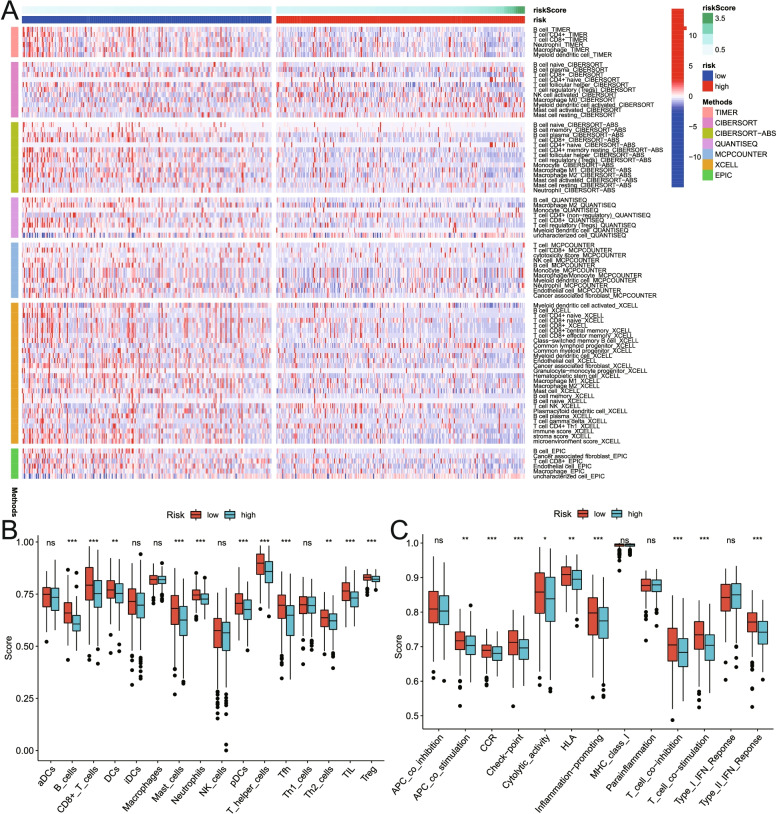
Correlation analysis of risk score and tumor immune infiltration in HNSCC. **A** The heat map showed the correlation between the risk score and immune cell infiltration. **B-C** The ssGSEA was used to calculate the degrees of immune cell infiltration and the expression patterns of immune-related molecules in the high- and low-risk groups. The results suggested that the degrees of immune cell infiltration and the expression levels of immune-related molecules in the low-risk group were higher than those of the high-risk group

TIMER, CIBERSORT, CIBERSORT-ABS, QUANTISEQ, MCPCOUNTER, XCELL, EPIC and other methods were used to measure the proportion of immune cells in HNSCC. The heat map was used to explore the correlation between risk score and immune cell infiltration. The results showed that the degree of immune cell infiltration in the low-risk score group was higher than that in the high-risk score group (Fig. 11A). In addition, the expression patterns of immune cells and immune-related molecules in the high- and low-risk groups were explored. The results showed that the risk score was correlated with the immune infiltrations of B cells, CD8 + T cells, DCs, Mast cells, neutrophils, pDCs, T helper cells, Tfh, Th2 cells, TIL and Treg (Fig. 11B). Meanwhile, the risk score was significantly correlated with the expression levels of immune-related molecules such as APC co inhibition, CCR, check-point, cytolytic activity, HLA, inflammation promoting, T cell co-inhibition, T cell co-stimulation and type II IFN response (Fig. 11C). In addition, we explored the impact of TP53 and TTN mutations on immune infiltration status. As shown in Figure S4A, in the TP53 group, the mutant group exhibited a lower degree of immune infiltration compared with the wild group. Meanwhile, as shown in Figure S4B, in the TTN group, the immune infiltration status of the mutant group and the wild group was not significantly different.

### Comprehensive analysis of genomic changes between high- and low-risk groups

**Fig. 12:**
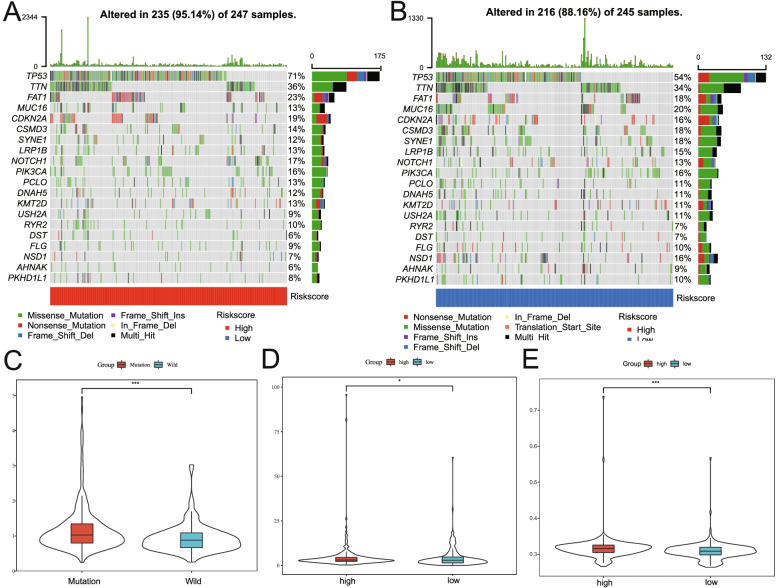
Comprehensive comparison of genomic changes between high- and low-risk groups. **A-B** Comparison of gene mutation rates between the high- and low-risk groups. **C** Comparison of the risk scores between the TP53 mutation and the TP53 wild groups. **D** Comparison of the mutation load status between in the high- and low-risk groups. **E** Comparison of the MSI status between the high- and low-risk groups. MSI, microsatellite instability

Using genetic mutation data from HNSCC patients in TCGA, genomic changes between high- and low-risk groups were compared. The results suggested that the high-risk group had a higher rate of gene mutations (Fig. 12A-B). At the same time, the risk score of the TP53 mutant group was higher than that of the TP53 wild group (*P* < 0.01, Fig. 12C). And the high-risk group had a higher gene mutation load than that of the low-risk group (*P* < 0.05, Fig. 12D). In addition, the MSI of the high-risk group was higher than that of the low-risk group (*P* < 0.01, Fig. 12E).

## Discussion

HNSCC is an aggressive malignant tumor with high incidence and poor prognosis. About 60% of HNSCC patients are already in advanced stages of local tumor when they are first diagnosed. After the failure of first-line treatment, the survival of patients dropped rapidly to about 3 months. Therefore, early diagnosis and treatment are of great significance to improve the clinical efficacy and survival of HNSCC patients. The insufficient predictive power of TNM staging shows the importance of screening new prognostic biomarkers for HNSCC. Therefore, this study aims to construct prognostic gene signature based on cancer hallmarks most relevant to the prognosis of HNSCC by comprehensive bioinformatics methods. Firstly, using ssGSEA and Cox-PH regression models, glycolysis and hypoxia were identified as cancer hallmarks most related to OS in HNSCC patients. All genes of the gene modules most related to glycolysis and hypoxia were extracted for further analysis. Then, WGCNA, COX univariate regression analysis, random forest algorithm and multiple combinations were used to construct this nine-gene signature (including RNF144A, STC1, P4HA1, FMNL3, ANO1, BASP1, MME, PLEKHG2 and DKK1) with prognostic value for HNSCC patients. Next, the prognostic value of the risk score based on the nine-gene signature was validated in the training set and the validation set. In addition, the correlations between the nine-gene signature and tumor immune infiltration profiles and genome changes were explored, respectively. Therefore, this nine-gene signature is an independent prognostic predictor of patients with HNSCC.

Previous studies revealed that biomarkers including mRNA, microRNA, lncRNA, DNA methylation and protein can contribute to the early diagnosis of HNSCC, the prediction of treatment response and the early monitoring of tumor recurrence [26]. For example, SERPINE1, PLAU and ACTA1 can be used as prognostic biomarkers of HNSCC [27]. PLA, CLDN8 and CDKN2A are also prognostic biomarkers for patients with HNSCC [28]. EGFR, CDK6 or CDK4 are associated with poor prognosis in HNSCC [29]. The overexpression of TMEM16A is related to the occurrence, proliferation and migration of tumor cells, and can be used as a potential biomarker of HNSCC [30, 31]. HILPDA, CD24, TCF3, SERPINE1, INHBA, P4HA2 and ACTN1 can be used to predict the response of locally advanced HPV-negative HNSCC patients receiving postoperative chemoradiation [32]. Moreover, miR-6508-5p, miR-210-5p, miR-4306 and miR-7161-3p are independent prognostic factors for HPV-negative HNSCC [33]. And miR-99a, miR-31, miR-410, miR-424 and miR-495 help predict the radiotherapy response of HNSCC patients [34]. FAM135B methylation is also an independent prognostic biomarker of HNSCC [35]. In addition, long non-coding RNAs such as AC024592.9, LINC00941, LINC01615 and MIR9-3HG, are associated with the prognosis of HNSCC [36]. IDO1 methylation can be used to predict the response of HNSCC patients to immune checkpoint inhibitors [37]. CDKN2A methylation is involved in the occurrence, progression and metastasis of HNSCC, and it is a potential diagnostic and prognostic biomarker for patients with HNSCC [38]. In addition, increased neutrophil-to-lymphocyte ratio is significantly associated with poor OS and DSS in HNSCC [39]. Besides, a previous study reported that Wang J. et al. explored the prognostic impacts of mutated genes in HNSCC and constructed a 6-gene risk model based on the hub genes [40]. The method used in our study to construct the prognostic model was different from that used by Wang J. et al. In our study, firstly, we explored the cancer hallmarks most associated with the prognosis of HNSCC patients using transcriptome profiling data and hallmark gene sets in the Molecular Signatures Database. The results showed that glycolysis and hypoxia were screened as major risk factors for the survival of HNSCC patients. Next, we used WGCNA to identify co-expression modules related to glycolysis and hypoxia. This method of screening cancer hallmarks-related genes based on network interaction is more in line with the actual situation of network regulation in the human body. Subsequently, we constructed a prognostic model based on univariate Cox analysis, random forest and multiple combinatorial screening. Therefore, this nine-gene signature which is significantly related to glycolysis and hypoxia adds new contents to the prognostic biomarkers of HNSCC.

The prognostic nine-gene signature established in this study included RNF144A, STC1, P4HA1, FMNL3, ANO1, BASP1, MME, PLEKHG2 and DKK1. As far as we know, DKK1 is involved in GPCR signal transduction and WNT ligand antagonist negative regulation of TCF-dependent signal transduction. PLEKHG2 participates in GPCR signal transduction. MME is related to peptidase activity and endopeptidase activity. BASP1 is related to the specific binding of protein domains. ANO1 is related to the activity of calcium-activated chloride channels in cells. FMNL3 is related to malignant melanoma and is involved in pathways including GPCR signal transduction and mitotic pre-metaphase. P4HA1 is related to oxidoreductase activity. STC1 is related to diseases such as fibrosarcoma and participates in ectodermal differentiation. RNF144A participates in pathways including protein metabolism and protein ubiquitination.

Previous studies suggest that increased DKK1 expression is associated with poor prognosis in HNSCC patients [41–44]. DKK1 methylation has prognostic value in HNSCC [45]. Increased expression of ANO1 could be used as a biomarker for distant metastasis and prognosis in HNSCC [46–49]. Increased expression of P4HA1 is associated with poor prognosis and increased risk of recurrence in HNSCC patients [21, 50–52]. P4HA1 plays a role in promoting tumor progression [53]. As one of the glycolysis-related genes, STC1 is also associated with the prognosis of HNSCC [54]. On the other hand, the impacts of the five hub genes (including RNF144A, FMNL3, BASP1, MME and PLEKHG2) on the prognosis of HNSCC patients have not yet been reported. Therefore, these results suggest that the mechanism of action of these nine hub genes in HNSCC deserves to be further explored.

In this study, we constructed a new nine-gene signature related to cancer hallmarks (including glycolysis and hypoxia). Survival analysis results confirmed that this nine-gene signature including RNF144A, STC1, P4HA1, FMNL3, ANO1, BASP1, MME, PLEKHG2 and DKK1 had good prognostic value for HNSCC patients. It is worth mentioning that recent studies had established some prognostic gene signatures for HNSCC patients as a supplement to traditional pathological staging. Comparing the survival prediction ability of previously reported gene signatures with the nine-gene signature will contribute to further assessing the prognostic values of these gene signatures. Therefore, we compared the survival prediction ability between the nine-gene signature constructed in this study and the previously reported two gene signatures. The results showed that the predictive ability of the nine-gene signature was better than the other two gene signatures within eight years, suggesting that this nine-gene signature was a good prognostic model in HNSCC.

The results of this study suggest that the risk score based on nine-gene signature is related to the immune cells infiltration status, the expression profiles of immune-related molecules and genome changes in HNSCC. Previous studies have shown that the immune microenvironment of HNSCC is characterized by changes in immune cells and immune checkpoints that make the balance of the immune environment beneficial to immune suppression, thereby allowing tumor cells to escape immune surveillance. Therefore, immunotherapy is expected to be a potentially beneficial supplement to the standard treatment of HNSCC [55–58]. Recent studies have shown that the immune infiltrating status is with prognostic value in HNSCC [59]. The prognosis of HPV-positive HNSCC patients is better than that of HPV-negative HNSCC patients, possibly due in part to the enhanced immune activation of the tumor microenvironment in HPV-positive HNSCC [60]. In addition, programmed death ligand 1 (PD-L1) is an immune checkpoint mainly located on the surface of tumor cells, and the positive expression of PD-L1 is associated with a better prognosis of HNSCC patients [61, 62]. Therefore, immunotherapies, especially those targeting the PD1 receptor or its ligand PD-L1, have shown significant efficacy in HNSCC [63]. In terms of genomic changes, tumor mutation burden (TMB) has been considered as a predictor of immune checkpoint inhibitors (ICIs) response. High TMB can identify HNSCC patients with poor prognosis after concurrent radiotherapy and chemotherapy [64]. PD-1 overexpression is associated with a good prognosis [65]. Currently known biomarkers predicting response to immune checkpoint inhibitors include PD-L1 expression, human papilloma virus infection, and microsatellite instability [66]. Therefore, the nine-gene signature constructed in this study were related to the degree of immune cell infiltration, the expression levels of immune-related molecules, and genomic changes. Meanwhile, we believe that two aspects should be considered to understand the results of the immune infiltration analysis. On the one hand, the predominant immune cells exhibited a higher degree of immune infiltration in the low-risk group. This may make the low-risk group have better prognosis. On the other hand, as shown in Fig. 11B, some immunosuppressive cells (such as T-reg cells) also had higher expression in the low-risk group. In addition, as shown in Fig. 11C, some immune-related molecules with opposite functions (such as T-cell co-stimulatory and T-cell co-inhibitory) were over-expressed at the same time in the low-risk group. We think that this is due to the complexity of the microenvironment. For example, Kubli, SP et al. proposed that the complexity of immune cell-cancer cell interaction is an important reason for the failure of tumor immunotherapy [67]. Besides, Feng, C et al., Prokhnevska, N et al. and Wierz, M et al. described the complexity of the tumor microenvironment in bladder cancer, prostate cancer, and chronic lymphocytic leukemia, respectively [68–70]. Taken together, we believe that the results of the immune infiltration analysis not only showed that the main immune cells exhibited a higher degree of immune infiltration in the low-risk group, but also provide some clues about the complexity of immune cell roles in HNSCC. These are the reasons why the immune infiltration status in HNSCC deserves to be further explored.

There are some limitations in this study. This study used a method of interactive verification among multiple independent datasets to verify the prognostic significance of the nine-gene signature. However, experimental validation is still an important step to further verify the prognostic value of this model. And the lack of experimental verification is the limitation of this study. In addition, this is a retrospective study, so the robustness and clinical usefulness of this nine-gene signature needs to be verified in prospective clinical trials.

In conclusion, this study identified a new prognostic gene signature related to glycolysis and hypoxia, including RNF144A, STC1, P4HA1, FMNL3, ANO1, BASP1, MME, PLEKHG2 and DKK1. Besides, a nomogram based on the nine-gene signature was constructed for clinical practice. This nine-gene signature can not only be used as a prognostic biomarker to help clinicians develop more personalized treatments for HNSCC patients, but also is expected to become a potential therapeutic target for HNSCC.

## Supplementary Information

**Additional file 1.**

## Data Availability

The datasets analyzed in the current study are available in the TCGA repository (http://cancergenome.nih.gov/) and the GEO (https://www.ncbinlm.nih.gov/geo/).
