# Supplementary material for: Transcriptome analysis reveals the prognostic and immune infiltration characteristics of glycolysis and hypoxia in head and neck squamous cell carcinoma
Source: BMC Cancer. 2022 Mar 31;22:352. doi: 10.1186/s12885-022-09449-9 (PMC8969218; doi:10.1186/s12885-022-09449-9)
Supplement: Supplementary file 1 — Additional file 1. [file 12885_2022_9449_MOESM1_ESM.docx]

**Supplementary Figures**


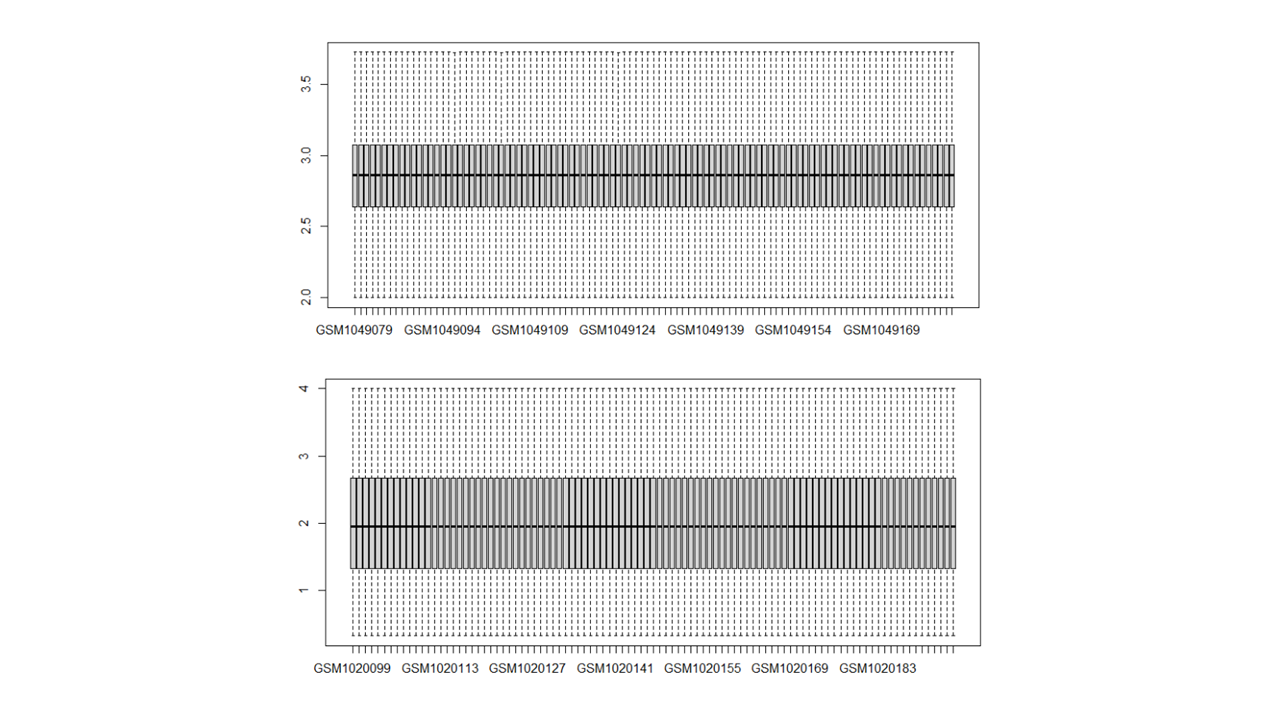


**Figure S1 Sample-normalized boxplots of two HNSCC cohorts from the GEO database.**


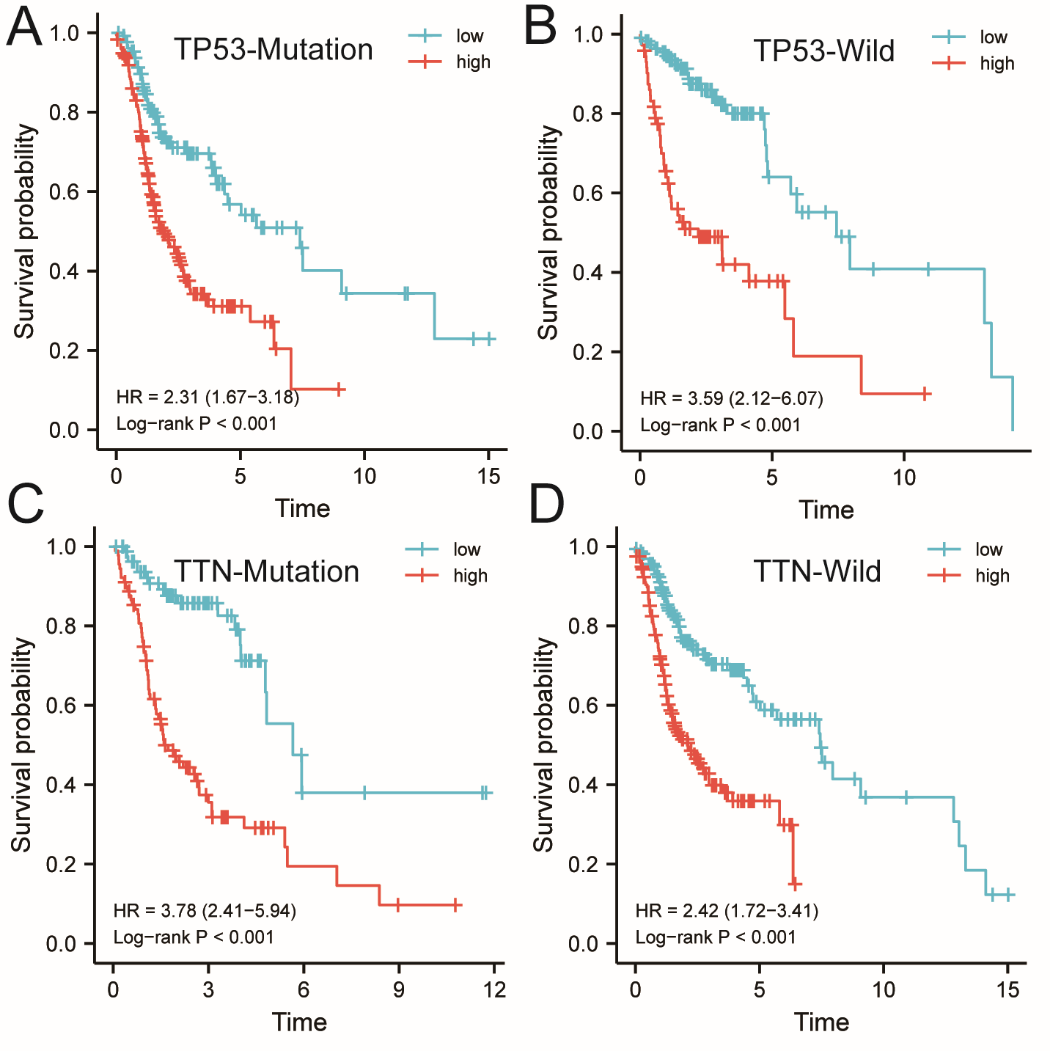


**Figure S2 Risk scores based on the nine-gene signature are markers of poor prognosis in HNSCC patients in various subgroups divided by gene mutations of TP53 and TTN.** (A-D) The nine-gene signature can distinguish high-risk patients in four subgroups (including TP53-Mutation, TP53-Wild, TTN-Mutation and TTN-Wild groups) based on gene mutations of TP53 and TTN.


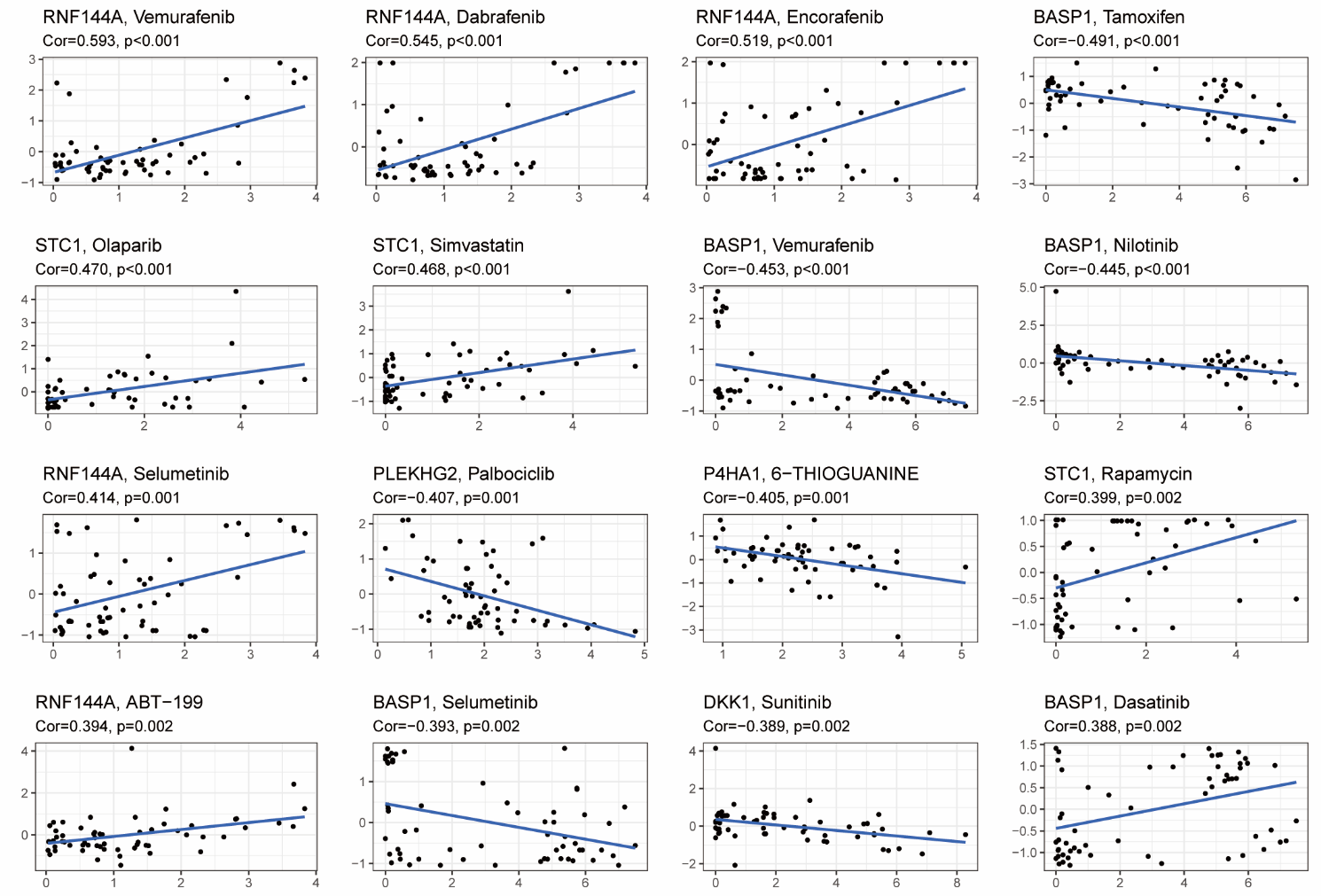


**Figure S3 Drug sensitivity analysis of nine hub genes.** RNF144A expression was positively correlated with the drug sensitivity of Vemurafenib, Dabrafenib, Encorafenib and ABT-199; BASP1 expression was negatively correlated with drug sensitivity of Tamoxifen, Vemurafenib, Nilotinib and Selumetinib; BASP1 expression was positively correlated with drug sensitivity of Dasatinib. STC1 expression was positively correlated with the drug sensitivity of Olaparib, Simvastatin and Rapamycin; PLEKHG2 was negatively correlated with drug sensitivity of Palbociclib, P4HA1 was negatively correlated with drug sensitivity of 6-THIOGUANINE. DKK1 was negatively correlated with drug sensitivity of Sunitinib. Cor, correlation coefficient.


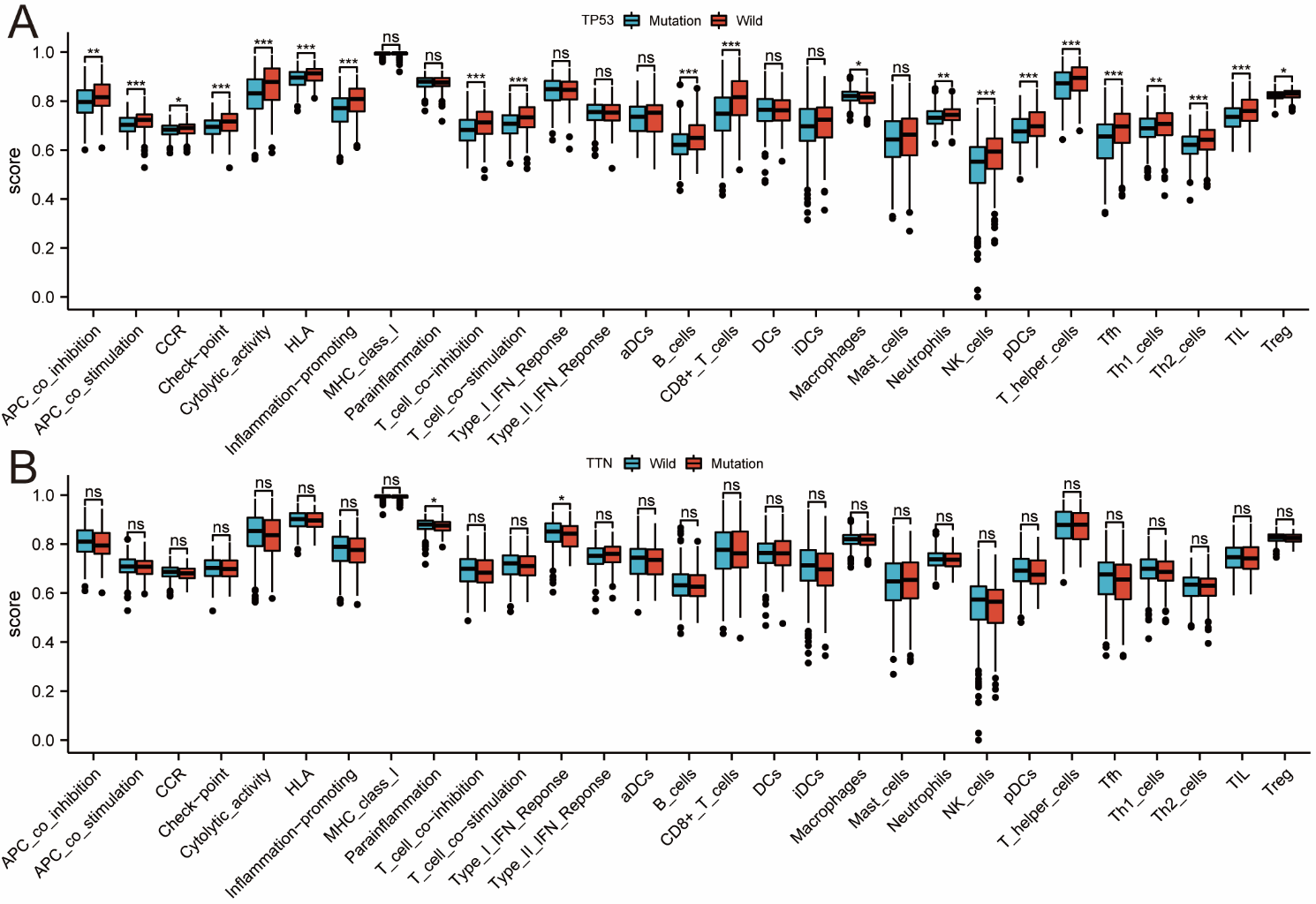


**Figure S4 Impact of TP53 and TTN mutations on immune infiltration status. (A)** In the TP53 group, the mutant group exhibited a lower degree of immune infiltration compared with the wild group. **(B)** In the TTN group, the immune infiltration status of the mutant group and the wild group was not significantly different.
